# Clinical and psychosocial context of HIV perinatally infected young mothers in Harare, Zimbabwe: A longitudinal mixed-methods study

**DOI:** 10.1371/journal.pone.0315299

**Published:** 2025-01-10

**Authors:** Zivai Mupambireyi, Victoria Simms, Webster Mavhu, Concilia Mutasa, Edward Matsikire, April Ricotta, Margaret Pascoe, Tinei Shamu, Beula Senzanje, Chiara Pierotti, Angela Mushavi, Nicola Willis, Frances M. Cowan

**Affiliations:** 1 Centre for Sexual Health & HIV Research Zimbabwe (CeSHHAR), Harare, Zimbabwe; 2 MRC International Statistics & Epidemiology Group, London School of Hygiene and Tropical Medicine, London, United Kingdom; 3 Department of International Public Health, Liverpool School of Tropical Medicine, Liverpool, United Kingdom; 4 Zvandiri, Harare, Zimbabwe; 5 Newlands Clinic, Harare, Zimbabwe; 6 UNICEF Zimbabwe, Harare, Zimbabwe; 7 Ministry of Health and Child Care, Harare, Zimbabwe; University of Washington, UNITED STATES OF AMERICA

## Abstract

**Background:**

The lives of adolescents and young people living with HIV (LHIV) are dominated by complex psychological and social stressors. These may be more pronounced among those perinatally infected. This longitudinal mixed-methods study describes the clinical and psychosocial challenges faced by HIV perinatally infected young mothers in Harare, Zimbabwe to inform tailored support.

**Methods:**

HIV perinatally infected young mothers were recruited in 2013 and followed up in 2019. In 2013, they completed a structured interview, clinical examination, psychological screening and had viral load and drug resistance testing. A subset completed in-depth interviews (n = 10). In 2019, they were re-interviewed and had viral load testing. Data were analyzed using STATA 15.0. and thematic analysis.

**Results:**

Nineteen mothers aged 17–24 years were recruited in 2013. Eleven (57.9%) were successfully recontacted in 2019; 3 had died, 2 had relocated and 3 were untraceable. In 2013, all 19 mothers were taking antiretroviral therapy (median duration 8 years, range 2–11 years) and median CD4 count was 524 (IQR 272). In 2013, eight mothers (42.1%) had virological failure (≥1000 copies/ml) (3 of whom subsequently died) and 7 (36.8%) had evidence of drug resistance. In 2019, the proportion with virological failure was 2/11 (18.1%). Six of 11 (54.5%) had switched to second line therapy. In 2013, 64.3% were at risk of common mental disorder and this risk was higher at follow-up (72.7%). Qualitative data highlighted three pertinent themes: HIV status disclosure, adherence experiences and, social and emotional support.

**Conclusions:**

Findings from this study underscore the significant clinical, social and psychological challenges faced by perinatally infected young mothers. The high rates of virological failure, drug resistant mutations, mental health issues and mortality observed in this population indicate the need for tailored and comprehensive health and support services to assist these young mothers.

## Introduction

Global HIV (95-95-95) targets are that 95% of all people living with HIV (LHIV] know their status, 95% of those with HIV are on sustained treatment and 95% of those on treatment are virally suppressed [1]. Several countries in sub-Saharan Africa (the epicenter of the HIV epidemic) [2], including Zimbabwe, are on track to achieve these targets [3]. Despite this, achievement of targets is not uniform across population groups. Adolescents and young people are less likely to be identified, on treatment and virally suppressed [3]. Adolescent girls and young women (AGYW, aged 15–24) are a particularly important group within this region; accounting for nearly one-quarter of all new HIV infections in 2022 [4].

AGYW pregnancy and birth rates within sub-Saharan Africa are the highest in the world [5]; early motherhood therefore occurs within the context of high HIV rates [6]. Pregnancy is a major life event accompanied by social, psychological and hormonal changes that can trigger depressive episodes with serious implications for both maternal and infant outcomes [7, 8]. Further, adolescent mothers LHIV are more likely than adults to transmit the virus to their infants [6, 9, 10]. In addition to managing their own HIV status, adolescent and young mothers LHIV need to also account for the physical, emotional and cognitive development of their children [10].

As in much of sub-Saharan Africa, perinatally infected (when HIV is passed on during pregnancy, childbirth or chest feeding) young mothers in Zimbabwe face risks that differ from those of mothers who acquired HIV later in life [11]. They may have cognitive impairment and poor psychological health [12]. They have often experienced HIV-related orphaning [13, 14] and, as a result, may have lacked a stable home environment or consistent parental role models [15]. This cohort born with HIV between 2000 and 2010 is now transitioning into adult care, becoming sexually active, and potentially getting pregnant [16].

A systematic review published in 2020 identified critical evidence gaps in sub-Saharan Africa in relation to adolescents LHIV [10]. These include a lack of primary data on their mental health and a dearth of research on their longitudinal health outcomes [10]. We attempt to fill in these gaps by using longitudinal data to describe the clinical and psychosocial challenges faced by a cohort of perinatally infected young mothers in Harare, Zimbabwe.

## Materials and methods

### Study setting and recruitment

We conducted the study among mothers aged 17–24 attending the Newlands HIV clinic and the Zvandiri program in Harare, the capital city of Zimbabwe. Harare’s population is around 2 million, with a disproportionate HIV burden [17]. Newlands clinic (www.newlandsclinic.org.zw) provides HIV prevention, counselling, treatment, care and support to around 8,000 under-privileged adults, children and adolescents LHIV [18]. Zvandiri (meaning ‘As I am’ in Shona (www.zvandiri.org), which was recommended by WHO [19, 20], UNAIDS [4] and PEPFAR [21] as a best practice program, is a theoretically-grounded, multi-component differentiated service-delivery model for children, adolescents, and young people LHIV [22–24]. Zvandiri has been scaled-up in Zimbabwe and adopted/adapted in 13 other African countries.

Recruitment of perinatally infected mothers involved a multi-stage process. Newlands clinic staff first identified them from their clinic records (which included their previous HIV history). This list of potential participants was then validated by Zvandiri staff using their electronic support group members’ database, before being handed over to the research team for the final recruitment phase. In 2013, thirty mothers were invited to participate in the study. Eligibility criteria included: awareness of HIV status, being a parent of ≥1 child below 5 years and able to provide informed consent. Exclusion criteria were: being too physically unwell to participate and acquiring HIV later in life (based on clinic records). The cohort was recruited from 25 November to 13 December 2013 and followed up six years later.

### Data collection

#### Baseline

At enrolment, participating mothers (n = 19) self-completed a questionnaire using Audio Computer-Assisted Self-Interviewing (ACASI), had a full clinical examination including WHO HIV staging. The WHO staging in 2013 categorized individuals based on clinical and immunological parameters into four stages: 1-Asymptomatic, 2-Mild symptoms, 3-Advanced symptoms, and 4-Severe symptoms or AIDS-defining conditions. By 2019, the WHO had shifted to the "treat all" strategy, recommending immediate antiretroviral therapy (ART) for all diagnosed with HIV, irrespective of clinical stage or CD4 count, to enhance outcomes and reduce transmission rates [25].

Participants were also screened for risk of common mental disorder (CMD) using the Shona Symptom Questionnaire (SSQ-14) by a certified psychiatrist. The SSQ-14 is a locally developed and validated 14-item indigenous CMD measure [26, 27]. Participants are asked if they have experienced a list of common mental health symptoms in the past week. Each of the 14 items are scored dichotomously as yes (1) or no (0). A score of ≥8/14 suggests risk of CMD (depression and/or anxiety) [26, 27]. Five mothers were not screened using SSQ-14 as they failed to attend the scheduled screening appointment, which was separate from the interview one.

Whole blood samples were collected from each participant for immunological assessment including CD4 and viral load testing. The samples were tested for viral load using COBAS Ampliprep®/COBAS Taqman® platforms and the HIV 1 version 2.1 kits, at the Newlands clinic laboratory. CD4 count was measured using BD Facscount (Becton Dickinson, Franklin Lakes, New Jersey, USA). When the viral load was ≥1000 copies/ml, an aliquot of the frozen plasma was sent to South Africa for a genotypic resistance test of the protease and reverse transcriptase genes, as resistance testing was unavailable in Zimbabwe at the time.

Ten mothers were purposively selected for in-depth interviewing; ensuring a range of factors (e.g., age, marital status and treatment history) which were considered important in shaping clinical and psychosocial experiences. Focusing on just 10 mothers was a deliberate strategy to ensure that the participants involved had a wide range of experiences, relevant to answering the research question, but also limited the scale of data collection so that detailed analyses could be conducted.

#### Follow-up

In 2019, an attempt was made to follow up all 19 mothers through the Zvandiri program and their baseline contact details. Eight (42.1%) mothers were lost to follow-up. All of those retained (n = 11; 58%), had recently transitioned from the Zvandiri adolescent support groups into a new program, Zvandiri Young Mentor Mothers (YMM) program. The YMM model trains and mentors young mothers LHIV as peer counsellors who are integrated within the national elimination of mother-to-child transmission of HIV program, and case manage other pregnant and chest feeding AGYW, 18–24 years, through to the end of chest feeding. Each YMM case manages their respective caseload, delivering peer-led counselling, monitoring and support through home visits, support groups, clinic visits and by phone. In this way, the YMMs deliver tailored care and support to vulnerable mother-baby pairs and partners who are often forgotten in programming [10].

Those retained completed a follow-up questionnaire using ACASI and had a full clinical examination and finger-prick blood sample taken for viral load testing using the NucliSENS EasyQ HIV-1 assay (BioMerieux, Inc., Madrid, Spain). Drug resistance testing was not conducted in 2019 due to resource constraints. Clinical records of those mothers who died between 2013 and 2019 were reviewed to ascertain cause of death.

Of the 11 retained mothers, five had been part of qualitative interviews at baseline and participated in a follow-up in-depth interview. All discussions took place in a private location in English or Shona (indigenous language) as preferred by the participant. They were audio-recorded and subsequently transcribed verbatim (and translated into English where necessary).

#### Data analysis

Quantitative data were analyzed using STATA 15.0. Descriptive summaries and frequencies of social, clinical and demographic characteristics were conducted. Qualitative data were uploaded, coded and summarized using a qualitative software package (NVivo 9.0, QSR International). We then conducted a phased process to systematically process the data, starting with data familiarization, including reading through the transcripts. Secondly, we created initial codes and categories to capture common topics in the data and categories relevant to the research questions. CM and ZM discussed the initial codes and agreed on their suitability. Thirdly, themes and sub-themes were generated from the initial codes. Fourthly, ZM reviewed the themes/sub-themes in consultation with an advisor (FMC) who gave her input on their validity. The themes/sub-themes were subsequently defined, named and used to provide context to qualitative data. Analysis of longitudinal data sought to identify specific trends.

### Ethical considerations

The study was reviewed and approved by the national ethics committee, the Medical Research Council of Zimbabwe at baseline (A/1755) and follow-up (A/2374). We obtained written informed consent from all participants including 17 year-old mothers, who are considered emancipated minors. Confidentiality assurances were given during the consent process. For confidentiality purposes, pseudonyms (fictitious names) were used where necessary.

## Results

### Quantitative findings

In 2013, mothers enrolled in the Zvandiri program (n = 30) were invited to participate. Nine were excluded from the study as they did not have perinatally acquired HIV based on their clinic records; two declined to participate. Nineteen mothers were recruited, 11 (57.9%) of whom were retained in 2019. In the interim, three mothers died of cryptococcal meningitis, pneumonia and an unknown cause; all had virological failure and two of three who died had CD4 counts <200 in 2013 (shown in Table 3). Two mothers had relocated to South Africa and three were lost to follow-up.

### Demographic characteristics of mothers

In 2013, mothers were aged 17–24 years; 11/19 (57.9%) had lost both parents, 3/19 (15.8%) had lost a mother and 2/19 (10.8%) had lost a father (Table 1). Three mothers (15.8%) had both parents alive. In 2013, 11/19 (57.9%) mothers were married while eight (42.1%) were separated. At the time of follow-up 6/11 (54.5%) were still married and mostly staying with their partners, 2/11 (18.2%) were divorced and 3/11 (27.3%) were widowed (Table 1).

**Table 1:** Demographic characteristics of mothers.

Characteristic			2013 (n = 19)	2019 (n = 11)
**Age (years)**		Median (range)	21 (17–24)	25 (23–30)
**Age at menarche (years)**		Median (range)	16 (13–19)	
**Age at sexual debut**		Median (range)	19 (16–23)	
**Age of HIV diagnosis awareness**		Median (range)	13 (9–20)	
**Age at ART initiation**		Median (range)	16 (9–22)	
**Duration on ART (years)**		Median (range)	8 (8–11)	17 (16–18)
**Orphanhood status**	Maternal orphans	N (%)	3 (16%)	3 (27%)
	Paternal orphans	N (%)	2 (11%)	3 (27%)
	Double orphans	N (%)	11 (58%)	5 (46%)
	Non orphans	N (%)	3 (16%)	0
**Marital status**	Married	N (%)	11 (58%)	6 (55%)
	Divorced /separated	N (%)	8 (42%)	2 (18%)
	Widowed	N (%)	0	3 (27%)
**School status**	Primary	N (%)	1 (5%)	
	Secondary	N (%)	17 (90%)	
	Certificate/Diploma/Degree	N (%)	1 (5%)	
**Employment status**	Employed	N (%)	2 (11%)	3 (27%)
	Self employed	N (%)	1 (5%)	3 (27%)
	Unemployed	N (%)	16 (84%)	5 (46%)

#### HIV history of mothers

At enrolment, the median age for HIV diagnosis was 13 years (range 9–20 years) (Table 1). All mothers were on ART, with median treatment duration of 8 years (range 8–11 years) (Table 1) and median CD4 count of 524 (IQR 272) (Table 2). Fourteen (73.7%) were on 1^st^ line therapy, taking Tenofovir+Lamivudine+Nevirapine. Five (26.3%) were on 2^nd^ line therapy. Of these, three were taking Tenofovir+Lamivudine+Lopinavir+Ritonavir, one was on Didanosine+Abacavar+Atazanavir+Ritonavir and the other was on Emtricibine+Tenofovir+Lopinavir+Ritonavir. In 2019, 5/11 (45.5%) of the mothers were still on first line regimens and 6/11 (54.5%) were on second line (Table 2). In 2013, 7/19 (36.8%) participants reported medication disengagement at some point. In 2019 only 2/11 (18.2%) reported ever having treatment interruption.

**Table 2:** Clinical and laboratory characteristics.

	2013						2019					
Study ID	CD4 count	Viral load	ART regimen	Current ART reg	Mental Health scores (SSQ)	Resistance to NRTIs	CD4 count	Viral load	ART regimen	Current ART reg	Mental Health scores (SSQ)	HIV status of child
**1**	1000	36	1^st^ line	Tenofovir- Lamivudine+ Nevirapine			1900	1318	1^st^ line	Tenofovir- Lamivudine+ Nevirapine	11	Negative
**2**	376	14040	1^st^ line	Tenofovir- Lamivudine+ Nevirapine	14	Yes	800	TND	2^nd^ Line	Abacavir+ Lamivudine+ Atazanavir/Ritonavir	2	Negative
**3**	849	36	1^st^ line	Tenofovir- Lamivudine+ Nevirapine	7		973	TND	1^st^ line	Tenofovir- Lamivudine+ Efavirenz	8	Negative
**4**	566	2919	1^st^ line	Tenofovir- Lamivudine+ Nevirapine	10	Yes	600	<839	2^nd^ line	Abacavir+ Lamivudine+ Atazanavir/Ritonavir	10	Negative
**5**	334	8917	1^st^ line	Tenofovir- Lamivudine+ Nevirapine	7	Yes	1900	TND	2^nd^ line	Abacavir+ Lamivudine+ Atazanavir/Ritonavir	7	Negative
**6**	597	36	1^st^ line	Tenofovir- Lamivudine+ Nevirapine	8		35	<839	1^st^ line	Tenofovir- Lamivudine+ Efavirenz	14	Negative
**7**	457	36	1^st^ line	Tenofovir- Lamivudine+ Nevirapine			1900	<839	2^nd^ line	Abacavir+ Lamivudine+ Atazanavir/Ritonavir	4	Negative
**8**	581	4388	1^st^ line	Tenofovir- Lamivudine+ Nevirapine	9	No	1900	<839	2^nd^ line	Tenofovir- Lamivudine + Atazanavir/Ritonavir	13	Negative
**9**	782	122	1^st^ line	Tenofovir- Lamivudine+ Nevirapine	6		99	TND	1^st^ line	Tenofovir- Lamivudine+ Efavirenz	14	Unknown
**10**	524	36	1^st^ line	Tenofovir- Lamivudine+ Nevirapine	11		652	<839	1^st^ line	Tenofovir- Lamivudine+ Efavirenz	10	Negative
**11**	24	498000	2^nd^ line	Tenofovir- Lamivudine+ Lopinavir-Ritonavir		Yes	1900	1318	2^nd^ line	Tenofovir- Lamivudine + Atazanavir/Ritonavir	9	Negative
**Not followed up**												
**12**	89	116500	1^st^ line	Tenofovir- Lamivudine+ Nevirapine	10	Yes	Died (2014)					
**13**	188	10640	1^st^ line	Tenofovir- Lamivudine+ Nevirapine	5	Yes	Died (2017)					
**14**	413	36	2^nd^ line	Emtricibine +Tenofovir + Lopinavi/-Ritonavir	6		Relocated					
**15**	988	36	1^st^ line	Tenofovir- Lamivudine+ Nevirapine	8		LTFU					
**16**	619	4080	2^nd^ line	Didanosine/Abacavir/Atazanavir/ritonavir	14	Yes	Died (2017)					
**17**	648	36	1^st^ line	Tenofovir- Lamivudine+ Nevirapine			LTFU					
**18**	382	36	2^nd^ line	Tenofovir- Lamivudine + Atazanavir/Ritonavir			Relocated					
**19**	597	36	2^nd^ line	Tenofovir- Lamivudine + Atazanavir/Ritonavir	8		LTFU					

Of the 19 mothers recruited in 2013, 14 (73.7%) had undergone WHO staging at diagnosis and of these, two were stage 1, five were stage 2, six were stage 3 and one was stage 4.

#### Viral suppression and drug resistance

In 2013, 8/19 (42.1%) had a viral load ≥1000 copies/ml. In 2019, 2/11 (18.2%) had virological failure (viral load ≥1000 copies/ml). Of the eight samples genotyped in 2013, seven had major drug resistance mutations and all seven had dual class resistance to NRTI and NNRTI. M184V and K65R were the most frequent NRTI-related mutations, while K103N, E138A and Y181C were the most frequent NNRTI-related mutations (Table 3).

**Table 3:** NRTI and NNRTI resistance mutations.

	NRTI Resistance Mutations										NNRTI									
ART Regimen	K65R	M184V	A62V	T215ST	T215Y	T215F	F77L	V75I	K70Q	Y115F	A98G	K103N	Y181C	V106M	E138A	V179E	G190A	V179T	A98AG	K101E
Tenolam + NVP		x			x							x					x			
ddI/ABC/ATV/r	x	x									x	x	x							
Tenolam + NVP	x	x	x	x									x							
Tenolam + NVP	x																			
missing	x													x	x	x				
Missing		x				x	x	x	x	x		x		x	x			x		
Missing		x				x									x		x		x	x
Tenolam + NVP	x	x	x										x							

### Mental health

At baseline, nine mothers were referred to a psychiatrist or psychologist as they were at risk of CMD (defined as scoring ≥8/14 on the SSQ). Six scored 8–10 suggesting risk of CMD and three scored ≥11 suggesting risk of severe CMD; five scored <8 and 5 missed the SSQ screening. At follow-up, CMD symptoms remained common with 8/11 (72.7%) scoring ≥8 on the SSQ-14. Of the three mothers with symptoms of severe CMD at baseline, one had died, one scored 10 and remained at risk of CMD while the third was no longer at risk (scored 2 in 2019).

### Clinical characteristics of infants

In 2013 and 2019, infants born to chest feeding mothers on ART were given daily Nevirapine (NVP) prophylaxis for six weeks from birth, while those receiving replacement feeding were given four to six weeks of daily NVP or twice daily azidothymidine (AZT) prophylaxis. At baseline, 19 infants were aged from two weeks to 40 months; eighteen babies had received NVP prophylaxis while one mother reported that her baby had not. Only 10/19 (52.6%) babies were on cotrimoxazole prophylaxis. No treatment interruptions were reported among infants put on nevirapine and cotrimoxazole.

Eighteen infants were up to date with their scheduled vaccinations, with only one child having missed vaccination and growth monitoring visits. Sixteen mothers (84.2%) reported that their babies had been tested for HIV and three (15.8%) reported that theirs were still too young (under six weeks) to be tested. Of the 16 babies that were reportedly tested, four (25.0%) mothers stated that they had not collected their baby’s result and of those with a result, all 12 (100%) tested HIV negative. We planned to follow up all 18 babies but only managed to reach 10 in 2019 (one child had been forcibly taken from the mother by her in-laws when she and her husband divorced). Of the ten children, all remained HIV negative, and we were unable to get information on children whose mothers died in the interim.

### Qualitative findings

We present qualitative findings to provide context to some of the data obtained quantitatively. We focus on three pertinent themes: HIV status disclosure, adherence experiences and, social and emotional support).

### HIV status disclosure

All mothers reported struggling to disclose their HIV status to male sexual partners and others, which affected medication adherence, antenatal care (ANC) (all but two attended ANC after 20 weeks, although they reported being in regular HIV care) as well as access to psychosocial services. Nearly all participants mentioned that they had wanted to disclose their HIV status to male partners before engaging in unprotected sex but could not do so. *‘I really wanted to tell him [spouse] before having sex*, *but I didn’t know how to tell him*. *I was afraid that he would “dump” me*. *I kept saying “I will tell him” and never did and then we had sex*, *and I became pregnant…’* (Chipo, 20 years). Fear of rejection by partner (and his family) was the main reason for not disclosing and the hesitancy generally persisted for six years.

Three mothers were, however, an exception as they reported disclosing to their male partners at some point, including before engaging in sex. *‘There was no way I was going to keep it a secret from him*, *so I said it’s better if I tell him now [whilst dating] so that we don’t “waste” each other’s time…’* (Tendai, 21 years). The three mothers reportedly received spousal support in the form of medication reminders and collection. They, however, mentioned pleading with their partners not to in turn, disclose to in-laws and significant others for fear that their status would be used against them. However, abuse was sometimes perpetrated by the male partner. A participant described how her husband became abusive and would mention her status in the presence of other family members. She had to move out of the matrimonial home, unable to tolerate the abuse. At follow-up, she had been diagnosed with depression and was receiving treatment.

### Adherence experiences

Perhaps unsurprisingly, non-disclosure of status impacted medication adherence. For example, in 2013 Nyasha had a high viral load despite being on second line treatment. She described how difficult it was to consistently adhere to her routine clinic visits and medication. *‘When I fell pregnant*, *I wondered what my husband and in-laws would say about my status*, *so I stopped collecting my drugs… I did not want to be seen taking them or even carrying them in my bag…’* (Nyasha, 22 years). Worrying about partner discovering pills and therefore, inadvertent disclosure, was a common theme.

Some mothers reported how treatment switches (i.e., from 1^st^ to 2^nd^ line) had bolstered adherence. A participant who had a viral load of 8,917 copies/mL in 2013 explained how being moved onto second line treatment changed her adherence behavior, leading to viral suppression.

*‘When I talked to you the first time, I had challenges with my medication, I would miss taking my pills and some days I would not take on time…. This changed when I was put on second line. The sister [nurse counsellor] told me that I was now on second line and that I must not miss because if the medication failed to work in my body, they wouldn’t have anything else to give me. They said that there was no other medication in Zimbabwe that they could help me with. I thought about it and decided that I would try by all means to adhere to my medication; since that day, I have had no challenges’* (Tatenda, 23 years).

This example highlights the potential positive role health-care workers involved in the care of young people LHIV could play in promoting adherence.

### Social and emotional support

Most of the participants reported receiving limited or no social support overall. A double orphan described how difficult it was for her to cope with motherhood initially. Her relatives were angry with her for falling pregnant out of wedlock and also, when she was on ART.

*‘When I gave birth, it was really difficult. I had no support at all, even from my own relatives. My uncles chased me away from home and they went to my boyfriend’s relatives shouting and demanding lobola [bride price]. I stayed with a distant relative who never bothered to help me with the baby and household chores even though I was nursing an operation [caesarean section], I struggled’* (Sekai, 19 years).

Another participant, also a double orphan, shared a similar story of neglect and social exclusion, where she was left to fend for herself and her newborn alone. Nearly all participants who had lost especially their mother felt that they would have received better support if she had been alive. They all mentioned the importance of having a “mother to support you soon after giving birth”.

At follow-up, however, all participants appreciated the social and emotional support from the newly established Zvandiri Young Mentor Mothers (YMM) program. Young mothers found participating in a bespoke program very helpful as they received tailored care and support, together with their children and partners. They described the YMM support group as a trusted social space to ask questions and discuss concerns they had regarding their babies and their own health.

*‘The YMM has empowered me with knowledge about living positively with HIV as a young mother. I now have friends who are also in the same situation as me, and they support me with information. Whenever I have a question about my daughter, I first ask my peers in our support group, and I receive amazing information. I now understand how I can be the best mother to my daughter’ (Natalie 18 years).*

Participants also described receiving adherence counseling during support group meetings. YMMs shared their experiences of living with HIV, particularly how to prevent HIV transmission to babies. Participants reported that this instilled the understanding that it was possible for babies to be HIV negative despite mothers LHIV, and they felt motivated and encouraged to adhere to treatment for their babies’ positive health outcomes.

## Discussion

We explored the clinical and psychosocial characteristics of perinatally infected young mothers. Study findings showed that these young mothers’ lives were particularly challenging as evidenced by disclosure issues, high virological failure and CMD, relationship dissolution and, limited social and emotional support. Six years on from early pregnancy, young mothers were still adapting to these challenges.

Voluntary disclosure of HIV status to partners continues to be a challenge for young mothers LHIV regardless of mode of transmission [28, 29]. As shown by this study, young people’s decision to disclose their HIV status is influenced by a number of social factors including fear of HIV-related stigma and discrimination and, rejection by partners [10, 29–31]. Our findings are in line with other research from sub-Saharan Africa where non-disclosure continues to be a public health challenge [30, 32–34]. Although a number of studies have shown the benefits of disclosure between partners [34, 35], how to ensure the disclosure process does not result in undesirable consequences remains a key consideration for interventions promoting disclosure of HIV positive status to the male partner [24, 28].

Baseline results showed relatively high rates of HIV drug resistance among the young mothers, resulting in the need to switch many to second-line treatment regimens. In a separate study among adolescents and young adults in Harare, despite enhanced adherence counselling, drug resistance among those failing first-line ART was notably high, with 97% exhibiting drug resistance mutations [36]. These findings underscore the urgent need for tailored interventions and innovative approaches to address the evolving needs of this group in order to ensure effective HIV management and positive long-term outcomes.

Suboptimal adherence to ART has long been identified as a major contributor to the development of HIV drug resistance among adolescents and young people LHIV [37, 38]. In our study, adherence was a significant challenge in 2013. A number of studies globally have shown that adherence to treatment gets worse in young people LHIV during the transition from childhood to adulthood, and tends to improve again thereafter [39–41]. Adherence problems in this population are predominately linked to social and relational challenges [28, 42]. For most mothers in our study, the need for acceptance by partners and in-laws outweighed the need for treatment compliance. The findings are in line with existing evidence which has shown that young people LHIV regardless of mode of transmission are under pressure to hide their status and medication from friends, family members and sexual partners [10, 43]. Studies have also shown that adherence and retention rates for women with HIV often decrease postnatally [44, 45]. Interventions targeting couples, such as the YMM program should continue to encourage openness among couples to strengthen support around medication adherence.

Our study findings highlight the link between clinical and psychosocial outcomes among young mothers LHIV, with data corroborating previous findings among the same population which showed a high mental health burden among mothers LHIV and how it affected viral suppression [46]. A study in South Africa also showed an increased mental health burden among adolescent mothers LHIV compared to other groups [8]. Collectively, these findings highlight the need for mental health support among adolescent and young mothers LHIV, regardless of mode of transmission. As YMMs may not be able to identify all these mental health needs and if they do, have to refer to healthcare workers, this points to some unmet need (for individual counseling) and suggests that there are some gaps in accessing professional counseling through the healthcare workers. Ways to routinely identify women with poor mental health at antenatal clinics are therefore urgently required. Community-based lay healthcare worker mental health interventions, such as the Friendship Bench [47] which has been widely scaled-up in Zimbabwe and elsewhere, presents a method of closing the gaps.

Consistent with previous studies [23, 42, 48], this study’s participants highlighted the critical role of support groups among young people LHIV. Research has shown that support groups are a useful resource for facilitating self-acceptance and restoring the confidence that may be lost once one is diagnosed with a highly stigmatised infection (HIV) [42, 48]. Similarly, in this study, young mothers described support groups as puncturing a tenacious sense of isolation and reducing their fear of the present and future implications of their HIV status. Despite, their important role, support groups for people LHIV in general and young people LHIV in particular, are characterized by intermittent or inconsistent funding, with the result that they are oftent run infrequently. We conclude that support groups are a useful resource for young people LHIV and therefore, should be supported by stable funding and held more frequently.

A strength of this study is that we used repeated measures to assess outcomes over time where that was possible. We also used a mixed-methods approach to triangulate data. Whilst quantitative data highlighted the magnitude of issues, qualitative data explored the context within which these trends occurred, highlighting the recognized value of combining quantitative and qualitative approaches within evaluations [49]. One significant limitation of the study is that it was conducted prior to the national integration of dolutegravir into routine HIV care in Zimbabwe. The quantitative data, while still relevant to the care of HIV-infected young women in Zimbabwe, may be considered somewhat old considering the rollout of highly potent and effective drugs in suppressing HIV, leading to faster viral load reduction. Nonetheless, adherence to dolutegravir-containing regimens is still crucial and we believe the findings are still important and relevant. The mental health findings also remain critical, underscoring the necessity for mental health interventions for young women LHIV. The relatively small sample size and the loss to follow-up are additional limitations. Despite these limitations, our data represent the experience of a select group of young mothers from Harare and provide a detailed insight into their lives and experiences, information critical for informing tailored interventions. Encouragingly, the YMM program has been cited as one of the promising interventions for adolescent and young mothers LHIV [10].

## Conclusions

Findings from this study underscore the significant clinical, social and psychological challenges faced by HIV perinatally infected young mothers. The high rates of virological failure, drug resistant mutations, mental health issues and mortality observed in this population indicate the need for more robust and tailored clinical management approaches. Beyond the medical concerns, qualitative findings highlighted the profound social and emotional burdens these young women bear, including HIV status disclosure, relationship difficulties, and limited support systems. Many of these psychosocial stressors appear to be linked to the young mothers’ early experiences of orphanhood, which can compound the challenges of navigating both HIV and parenthood simultaneously. To comprehensively address the multifaceted needs of this vulnerable population, a holistic model of care is warranted. This should encompass not only specialized clinical services, but also integrated psychosocial support, counseling, and community-based support. Continued research and program development in this area are crucial to ensuring these young women receive the comprehensive support necessary to thrive.
